# Women Leaders and Policy Compliance during a Public Health Crisis

**DOI:** 10.1017/S1743923X20000604

**Published:** 2020-12

**Authors:** Nichole M. Bauer, Jeong Hyun Kim, Yesola Kweon

**Affiliations:** 1 Louisiana State University; 2 Louisiana State University; 3 Utah State University

**Keywords:** COVID-19, female leaders, policy compliance, survey experiment

## Abstract

How does the gender of a political leader affect policy compliance of the public during a public health crisis? State and national leaders have taken a variety of policy measures to combat the COVID-19 pandemic, with varying levels of success. While many female leaders have been credited with containing the spread of COVID-19, often through implementing strict policy measures, there is little understanding of how individuals respond to public health policy recommendations made by female and male leaders. This article investigates whether citizens are more willing to comply with strict policy recommendations about a public health issue when those recommendations are made by a female leader rather than a male leader. Using a survey experiment with American citizens, we compare individuals’ willingness to comply with policy along three dimensions: social distancing, face covering, and contact tracing. Our findings show that a leader's gender has little impact on policy compliance in general during the pandemic. These findings carry important implications for successful crisis management as well as understanding how a crisis in a nonmasculine issue context influences the effectiveness of a leader's ability to implement measures to mitigate the crisis.

How does the gender of a political leader affect policy compliance of the public during a public health crisis? Prior research suggests that voters prefer strong and aggressive leaders during crises (Gadarian 2010). While most studies focus on the effects of a leader's gender during crises that fit into “masculine” issue areas such as national security (Gadarian 2010; Holman, Merolla, and Zechmeister 2016; Simas 2020), there is little understanding of how crises in “feminine” issue areas such as public health affect evaluations of leaders. This knowledge gap affects our understanding of how individuals respond to female and male leaders working to mitigate a crisis. The results of this study are particularly important as a number of news outlets note that women lead the countries that have been most successful at limiting the spread of COVID-19 (Anderson and Auxier 2020; Henley and Roy 2020).

Using a survey experiment of Americans, we compare individuals’ willingness to comply with the COVID-19 policy recommendations regarding social distancing, face coverings, and contact tracing made by female leaders relative to those by male leaders. We examine how policy compliance varies based on the gender of the leader as well as the leader's partisanship.

Our findings show that a leader's gender has little impact on policy compliance in general during the pandemic. As the policy recommendations become more personally invasive, such as engaging in contact tracing, compliance weakly increases when the recommendation comes from a copartisan female leader, while a message from a copartisan male leader has no effect. Our findings that voters will comply with female leadership during a health crisis stand in contrast to previous findings showing that voters have more favorable attitudes toward strong male leadership during a crisis.

## GENDERED LEADERSHIP AND POLICY COMPLIANCE DURING A PUBLIC HEALTH CRISIS

In times of crisis, threatened individuals seek out strong, charismatic, and hawkish leadership (Gadarian 2010; Merolla, Ramos, and Zechmeister 2007). This tendency implies that voters are less likely to view women as competent at dealing with a national security crisis compared with men (Holman et al. 2019), and thus they more likely to comply with policy guidelines from male leaders.^1^

While the literature mostly focuses on crises in masculine issue areas, public health issues are typically considered to be feminine issue areas (Kaufman and Petrocik 1999; Schneider 2014). For typical women's issues, voters tend to rate female politicians’ competency higher than male politicians (Herrnson, Lay, and Stokes 2003; Huddy and Terkildsen 1993). A key difference between the crisis spurred by the pandemic and other crises, such as a national security crisis, is that the former calls for leaders to display feminine traits, such as compassion, rather than masculine traits, such as strength (Johnson and Williams, forthcoming).

Perceived competency of a female leader during the current pandemic could enhance the credibility of policy recommendations made by a female leader. This leads to an expectation that citizens’ willingness to comply with the policy measures that most public health experts say are critical to limiting the spread of the virus will increase when they are made by a female leader relative to a male leader.

However, there is ample evidence that voters are more likely to exhibit gender-motivated biases toward female political leaders when they belong to the *other* political party (Bauer 2017; Ditonto 2017; Krupnikov and Bauer 2014). Individuals may be most resistant to following the policy guidelines of female leaders when those leaders belong to the *opposing* political party.

## RESEARCH DESIGN AND METHODS

Our experiment used a 2 *×* 2 *×* 2 design in which we vary (1) the governor's gender (female/male), (2) policy recommendations (strong/no recommendations), and (3) relative partisanship (in-partisan/out-partisan). We conducted our study through Lucid, a survey sampling company that used a national convenience sample of adults in the United States, with a total size of *N* = 811. We include descriptive data on our sample in Table A1 in Appendix C in the supplementary materials online and more detailed explanations of the survey experiment in Appendix A. A total of 697 respondents (86%) passed the manipulation check.^2^ See Appendix B2 for the manipulation question wording. The balance test shows that the data are well balanced across key individual attributes (Appendix D).

The treatment used a press release explaining that the governor was moving to Phase 2 of reopening the economy during the pandemic. In the press release, the governor makes a *strong policy recommendation* that residents wear face coverings and social distance in public and says that the state will implement a contact tracing program. The second press release includes *no policy recommendation* from the governor. We framed the press release around the process of reopening the economy and included no explicit information about the spread of COVID-19 infection in that particular state. Including the no policy recommendation is theoretically important because it lets us gauge people's willingness to participate in mitigation efforts without direct guidance from a political official. We developed the treatment by reviewing the press releases from governors, at the time we developed the treatment in early June 2020, in states such as California, Louisiana, Michigan, and Ohio. See Appendix B1 for the full text of the treatment.

The key outcome variable, *compliance willingness*, is measured as respondents’ willingness to follow three policy measures with varying levels of restrictiveness on a 4-point scale. While the use of a hypothetical governor in a state where the respondent does not live could limit our ability to make inferences about people's actual behaviors, we prompt individuals to think about their own willingness to wear a face covering, social distance, and participate in contact tracing in response to the treatment they read; this is a standard protocol and design in survey experiments (Mutz 2011). See Appendix B3 for the outcome questions. For our empirical strategy, we use one-sided independent samples *t*-tests examining differences in compliance willingness among four groups.

## FINDINGS

We compare policy compliance across the strict policy treatment condition first among in-partisans and then among out-partisans. Then, we conduct comparisons separately for the female governor and the male governor from the no policy condition to the strict policy condition for the in-partisans and for the out-partisans. Together, these sets of comparisons offer the most rigorous tests for whether citizens are willing to listen to female leaders or reject their advice during a major national crisis.

**Figure 1.**
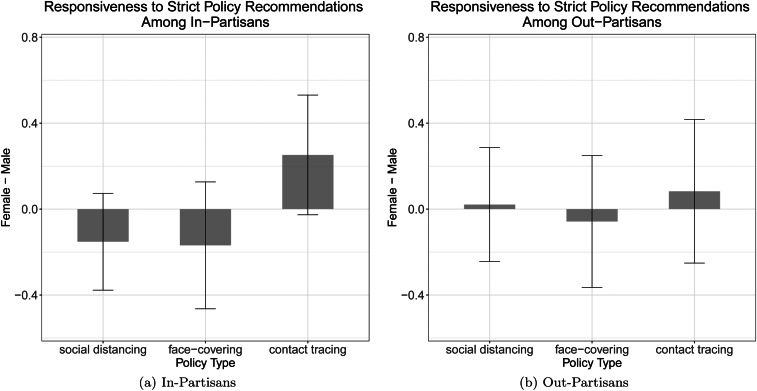
Gender difference in responsiveness to strict policy recommendations. In each panel, solid lines with error bars indicate 95% confidence intervals.

Figure 1 displays the mean differences in compliance willingness between female and male leaders who issue strict recommendations along with 95% confidence intervals. We display the results separately by in-partisan (a) and out-partisan groups (b). The higher value indicates more responsiveness to a female governor's recommendation.

The results suggest that there are no statistically significant gender differences in responsiveness to wearing a face covering and social distancing between in-partisans and out-partisans. For contact tracing, however, we find that respondents were more responsive to a female governor's recommendation compared with a male governor's. This difference was statistically significant at *p <* .10.

Although contact tracing plays a vital role in curbing the spread of the virus (Klinkenberg, Fraser, and Heesterbeek 2006), American citizens are wary of the government's use of tracing tools. In a recent survey in the United States, 48% of the respondents said it is unacceptable for the government to track people through their cell phones (Anderson and Auxier 2020). Since voters tend to view female officeholders as more ethical and more principled than male officeholders (Barnes and Beaulieu 2014; Dolan 2018), a female governor's recommendation to engage in contact tracing could mitigate people's concerns that the government might misuse its tracing tools, promoting willingness to comply with the policy.

Next, we investigate the differences in policy compliance between strict and no policy recommendations by a leader of the same gender and between female and male leadership. This analysis enables us to evaluate (1) how a governor's strong recommendation influences policy compliance willingness and (2) how that effect might differ by the leader's gender.

**Figure 2.**
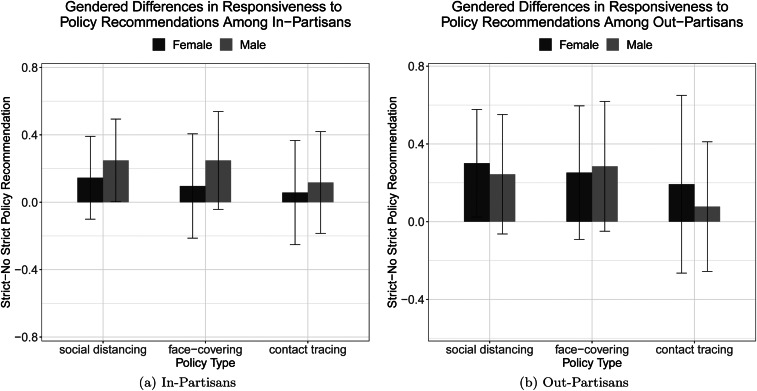
Gendered difference in responsiveness to policy recommendations. In each panel, solid lines with error bars indicate 95% confidence intervals.

In Figure 2, we report the differences in means between the strict and no strict policy recommendation conditions, disaggregated by governor gender. Our findings show some limited evidence of gendered effects. As shown in the left panel, strict policy recommendations by a female in-partisan leader have little impact on policy compliance. Respondents who received a strict recommendation from an in-partisan male governor displayed more willingness to maintain social distancing than those who received no recommendation by the same male governor. The effect size was roughly 24% of a standard deviation of the outcome measure. Similarly, a male governor's strict recommendation increased policy compliance with face-covering in public spaces, though the difference was only significant at the *p <* 0.1 level.

The right panel suggests that a governor's strong policy recommendations do not have a big impact on out-partisan voters’ compliance willingness. One exception was the effect on social distancing: a female governor's strict recommendations increased willingness to maintain social distancing, while a male governor's recommendations did not have the same effect.

## IMPLICATIONS

Our results show that citizens express policy compliance similarly toward the recommendations made by a female governor and a male governor during a pandemic. One may be concerned that conducting a study about COVID-19 in the middle of the pandemic makes it possible that preexisting attitudes about wearing a mask or social distancing affected how people responded to our treatments. Nevertheless, we find these results encouraging, especially considering a popular media narrative suggesting more resistance to female governors during the COVID-19 crisis. This study opens up the door for future work on the gendered nature of political leadership during a pandemic, such as whether women receive different types of evaluations on other leadership metrics, such as favorability, compared to men when they put in place policies to mitigate the crisis. Moreover, women may receive more gendered forms of criticism compared to men even if individuals are just as willing to follow mask recommendations. Our study suggests that the majority of citizens are willing to follow the advice and recommendations of female and male leaders.

## Footnotes

1. Some research suggests that the threat of a security crisis can lead to an increase in preferences for female leaders (Brown, Diekman, and Schneider 2011).

2. The reported analysis focuses on respondents who passed the manipulation check.

## Supplementary material

For supplementary material accompanying this paper visit https://doi.org/10.1017/S1743923X20000604. click here to view supplementary material

## References

[ref1] Anderson, Monica, and Brooke Auxier. 2020 “Most Americans Don't Think Cellphone Tracking Will Help Limit COVID-19, Are Divided on Whether It's Acceptable.” Pew Research Center, April 16. https://www.pewresearch.org/fact-tank/2020/04/16/most-americans-dont-think-cellphone-tracking-will-help-limit-covid-19-are-divided/-on-whether-its-acceptable/ (accessed August 27, 2020).

[ref2] Barnes, Tiffany D., and Emily Beaulieu. 2014 “Gender Stereotypes and Corruption: How Candidates Affect Perceptions of Election Fraud.” Politics & Gender 10 (3): 365–91.

[ref3] Bauer, Nichole M. 2017 “The Effects of Counterstereotypic Gender Strategies on Candidate Evaluations.” Political Psychology 38 (2): 279–95.

[ref4] Brown, Elizabeth R., Amanda B. Diekman, and Monica C. Schneider. 2011 “A Change Will Do Us Good: Threats Diminish Typical Preferences for Male Leaders.” Personality and Social Psychology Bulletin 37 (7): 930–41. 21467539 10.1177/0146167211403322

[ref5] Ditonto, Tessa. 2017 “A High Bar or a Double Standard? Gender, Competence, and Information in Political Campaigns.” Political Behavior 39 (2): 301–25.

[ref6] Dolan, Kathy. 2018 Voting for Women: How the Public Evaluates Women Candidates. New York: Routledge.

[ref7] Gadarian, Shana Kushner. 2010 “Foreign Policy at the Ballot Box: How Citizens Use Foreign Policy to Judge and Choose Candidates.” Journal of Politics 72 (4): 1046–62.

[ref8] Henley, John, and Eleanor Ainge Roy. 2020 “Are Female Leaders More Successful at Managing the Coronavirus Crisis?” *The Guardian*, April 25. https://www.theguardian.com/world/2020/apr/25/why-do-female-leaders-seem-to-be-more-successful-at-managing-the-coronavirus-crisis (accessed August 27, 2020).

[ref9] Herrnson, Paul S., J. Celeste Lay, and Atiya Kai Stokes. 2003 “Women Running ‘as Women’: Candidate Gender, Campaign Issues, and Voter-Targeting Strategies.” Journal of Politics 65 (1): 244–55.

[ref10] Holman, Mirya R., Jennifer L. Merolla, and Elizabeth J. Zechmeister. 2016 “Terrorist Threat, Male Stereotypes, and Candidate Evaluations.” Political Research Quarterly 69 (1): 134–47.

[ref11] Holman, Mirya R., Jennifer L. Merolla, Elizabeth J. Zechmeister, and Ding Wang. 2019 “Terrorism, Gender, and the 2016 U.S. Presidential Election.” Electoral Studies 61: 1–8.

[ref12] Huddy, Leonie, and Nayda Terkildsen. 1993 “Gender Stereotypes and the Perception of Male and Female Candidates.” American Journal of Political Science 37 (1): 119–47.

[ref13] Johnson, Carol, and Blair Williams. Forthcoming “Gender and Political Leadership in a Time of COVID.” Politics & Gender. 10.1017/S1743923X2000029X.

[ref14] Kaufman, Karen M., and John R. Petrocik. 1999 “The Changing Politics of American Men: Understanding the Sources of the Gender Gap.” American Journal of Political Science 43 (3): 864–87.

[ref15] Klinkenberg, Don, Christophe Fraser, and Hans Heesterbeek. 2006 “The Effectiveness of Contact Tracing in Emerging Epidemics.” PloS one 1(1). 10.1371/journal.pone.0000012 PMC1762362 17183638

[ref16] Krupnikov, Yanna, and Nichole M. Bauer. 2014 “The Relationship between Campaign Negativity, Gender, and Campaign Context.” Political Behavior 36 (1): 167–88.

[ref17] Merolla, Jennifer L., Jennifer M. Ramos, and Elizabeth J. Zechmeister. 2007 “Crisis, Charisma, and Consequences: Evidence from the 2004 US Presidential Election.” Journal of Politics 69 (1): 30–42.

[ref18] Mutz, Diana C. 2011 Population-Based Survey Experiments. Princeton, NJ: Princeton University Press.

[ref19] Schneider, Monica C. 2014 “The Effects of Gender-Bending on Candidate Evaluations.” Journal of Women, Politics & Policy 35 (1): 56–77.

[ref20] Simas, Elizabeth N. 2020 “But Can She Make America Great Again? Threat, Stability, and Support for Female Candidates in the United States.” Political Behavior. Published online April 3. 10.1007/s11109-020-09607-4.

