# Supplementary material for: Women Leaders and Policy Compliance during a Public Health Crisis
Source: Politics & Gender. 2020 Dec;16(4):975–82. doi: 10.1017/S1743923X20000604 (PMC7853737; doi:10.1017/S1743923X20000604)
Supplement: Supplementary file 1 [file S1743923X20000604sup001.pdf]

Online Appendix for Women Leaders and Policy Compliance during a Public Health Crisis

A. Details about Survey Experiment

We use a pre-registered survey experiment to examine the effect of gendered leadership on policy compliance.[1] In our experiment, the governor's name and profile photo were used to indicate the governor's gender. Photos are pretested, and female and male photos are comparable in other determinants such as perceived age, education level.

In order to have balanced samples of respondents across in-partisan and out-partisan conditions, we first blocked the respondents into Democrats and Republican groups, and then respondents in each group were randomly assigned to either a Democratic or Republican governor condition. For 168 respondents who indicate themselves as independents, we followed the strategy of past scholarship and asked participants to select a party they lean more closely toward (Krupnikov and Bauer 2014). All respondents, after receiving a treatment, were asked a manipulation check to ensure that the treatment is effective, and approximately 86% of respondents (N=697) passed the manipulation check. Among these 697 respondents, 377 were assigned to in-partisan conditions, and the remaining 320 respondents were assigned to out-partisan conditions.

Out experimental conditions include: 1) co-partisan female leader with strict policy recommendations; 2) co-partisan female without policy recommendation; 3) out-partisan female leader with strict policy recommendations; 4) out-partisan female without policy recommendation; 5) co-partisan male leader with strict policy recommendations; 6) co-partisan male without policy recommendation; 7) out-partisan male leader with strict policy recommendations; 8) out-partisan male without policy recommendation.

1 The pre-registration is available at: https://aspredicted.org/blind2.php

B. Survey Questions

B1. Treatments

* Below, we are only exhibiting the examples of a democratic governor's press releases. However, in the survey, We randomized the partisanship of a governor in the treatment.

1. Female + Democratic + Strong Policy Recommendations

We are interested in better understanding how people evaluate leaders during times of crisis. On the next page, we'd like you to read a press release from **Democratic Governor Carol Hartley** and then we'll ask you some questions.

Governor Carol Hartley Announces Plans for the State

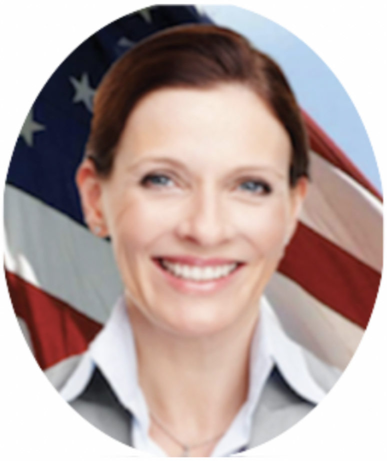

June 2020

Democratic Governor Carol Hartley today announced that the state will move to the next phase of the state's ongoing plan to reopen. Under this phase, restaurants may increase their occupancy to 50% and beauty salons, bowling alleys, bars, and youth sports may open with restricted occupancy levels.

Governor Carol Hartley **strongly recommends** that all citizens of the state that go out in public continue to maintain social distancing and wear a mask. Governor Hartley said the state **will also** implement full-scale testing for citizens and contact tracing to control the spread of the virus.

"We will continue to safely and strategically reopen the state. To move forward, we need to restore the state's economy and businesses." said Governor Carol Hartley.

2. Male + Democratic +Strong Policy Recommendations

We are interested in better understanding how people evaluate leaders during times of crisis. On the next page, we'd like you to read a press release from **Democratic Governor Chris Hartley** and then we'll ask you some questions.

Governor Chris Hartley Announces Plans for the State

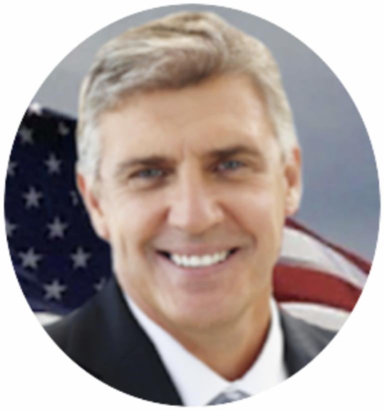

June 2020

Democratic Governor Chris Hartley today announced that the state will move to the next phase of the state's ongoing plan to reopen. Under this phase, restaurants may increase their occupancy to 50% and beauty salons, bowling alleys, bars, and youth sports may open with restricted occupancy levels.

Governor Chris Hartley **strongly recommends** that all citizens of the state that go out in public continue to maintain social distancing and wear a mask. Governor Hartley said the state **will also** implement full-scale testing for citizens and contact tracing to control the spread of the virus.

"We will continue to safely and strategically reopen the state. To move forward, we need to restore the state's economy and businesses." said Governor Chris Hartley.

3. Female + Democratic + No Policy Recommendations

We are interested in better understanding how people evaluate leaders during times of crisis. On the next page, we'd like you to read a press release from **Democratic Governor Carol Hartley** and then we'll ask you some questions.

Governor Carol Hartley Announces Plans for the State

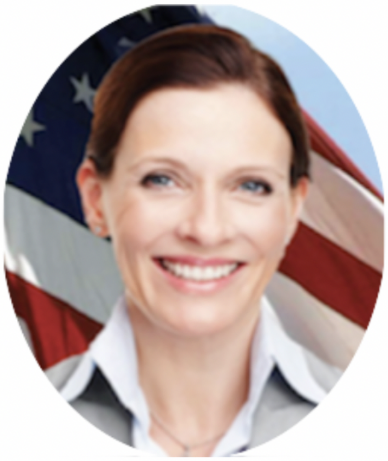

June 2020

Democratic Governor Carol Hartley today announced that the state will move to the next phase of the state's ongoing plan to reopen. Under this phase, restaurants may increase their occupancy to 50% and beauty salons, bowling alleys, bars, and youth sports may open with restricted occupancy levels.

Governor Carol Hartley is **not making any recommendations** about whether the public should maintain social distancing and wear a mask in public. Governor Hartley said the state **will not be implementing** full-scale testing for citizens and contact tracing to control the spread of the virus.

"We will continue to safely and strategically reopen the state. To move forward, we need to restore the state's economy and businesses." said Governor Carol Hartley.

4. Male + Democratic + No Policy Recommendations

We are interested in better understanding how people evaluate leaders during times of crisis. On the next page, we'd like you to read a press release from **Democratic Governor Chris Hartley** and then we'll ask you some questions.

Governor Chris Hartley Announces Plans for the State

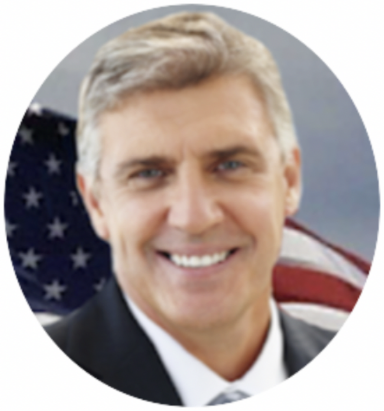

June 2020

Democratic Governor Chris Hartley today announced that the state will move to the next phase of the state's ongoing plan to reopen. Under this phase, restaurants may increase their occupancy to 50% and beauty salons, bowling alleys, and youth sports may open with restricted occupancy levels.

Governor Chris Hartley **is not making any recommendations** about whether the public should remain social distancing or wear a mask in public. Governor Hartley said the state **will not** be implementing full-scale testing for citizens and contact tracing to control the spread of the virus.

"We will continue to safely and strategically reopen the state. To move forward, we need to restore the state's economy and businesses." said Governor Chris Hartley.

B2. Manipulation Check Question For All Respondents

Q1. Based on your best recollection, was the politician you read about a man or a woman?
1. Man
2. Woman
3. Can't Recall

Q2. Based on your best recollection, what was the party identification of the politician you read about at the beginning of the study?
1. Democrat
2. Republican
3. Independent
4. Non-partisan

B3. Post-Treatment Questions

Q1. Policy compliance: Face-covering

Given the press release by Governor [Carol/Chris] Hartley, how likely are you to wear a face-covering in public spaces?

1. Very unlikely
2. Somewhat unlikely
3. Somewhat likely
4. Very likely

Q2. Policy compliance: Social distancing

Given the press release by Governor [Carol/Chris] Hartley, how likely are you to maintain strict social distancing in public spaces

1. Very unlikely
2. Somewhat unlikely
3. Somewhat likely
4. Very likely

Q3. Policy compliance: Contact tracing

Given the press release by Governor [Carol/Chris] Hartley, how willing are you to engage in contact tracing to better fight Covid-19?

1. Very unwilling
2. Somewhat unwilling
3. Somewhat willing
4. Very willing

B4. Pre-treatment Covariates

Q1. Gender
What is your gender?
1. Female
2. Male
3. Other

Q2. Age
What is your age?

Q3. Race
What is your race?
1. White
2. Black or African-American
3. American Indian or Alaska Native
4. Hispanic or Latino
5. Asian
6. Native Hawaiian or other Pacific Islander
95. Other [Please specify]

Q4. Education
What is the highest education that you have attained?
1. Less than high school (no high school diploma)
2. High school graduate
3. Community or junior college graduate
4. Four year college graduate
5. Some postgraduate education
6. Graduate or professional degree

Q5. State of residence
Choose the state of your current residence

Q6. Household income
What is your annual household income before taxes?
1. Under $5,000
2. $5,000-9,999
3. $10,000-12,499
4. $12,500-14,999
5. $15,000-17,499
6. $17,500-19,999

Q7. Employment status
What is your current employment status?
1. Full-time job
2. Part-tome job
4. Unemployed
5. Retired
6. Permanently disabled
7. Homemaker
8. Student

Q8. Medical service workers

Do you work in a medical service sector?
1. Yes
2. No
3. Don't know

Q9a. Partisanship
Which party do you support?
1. Republican party [Go to Q10]
2. Democratic party [Go to Q10]
3. Independent or other [Go to Q9b]

Q9b. Partisanship (Only for those who answered #3 in Q9b)
Which party do you feel closer to?
1. Republican party
2. Democratic party
3. No party feeling closer to

Q10. Incumbent vote
Did you vote for Donald Trump in the 2016 presidential Election?
1. Yes
2. No
3. Did not vote

Q11. Interest in politics
How much interested are you in politics?
1. Not interested at all
2. Little interested
3. Somewhat interested
4. Very interested

C. Summary Statistics

Table A1: Summary Statistics of Demographic Variables

	Min	Median	Mean	Max	Std. Dev	NA
Age	18	46	46.5	86	17.20	0
Female	0	1	0.536	1	0.499	7
Education	1	4	3.701	6	1.406	4
White	0	1	0.725	1	0.447	0

Table A2: Summary Statistics of Outcome Variables (Compliance Willingness)

	Min	Median	Mean	Max	Std. Dev	NA
Social Distancing	1	4	3.325	4	0.890	21
Face-Covering	1	4	3.125	4	1.057	8
Contact Tracing	1	3	2.885	4	1.044	1

Table A3: Means of Compliance Willingness by Respondent Party ID

	Democrats (N=352)	Republicans (N=345)	Difference in Means (p-value)
Social Distancing	3.356	3.295	0.061 (0.371)
Face-Covering	3.219	3.029	0.190 (0.018)
Contact Tracing	3.045	2.721	0.325 (0.000)

Table A3 reports the means of compliance willingness measures by respondent party ID. In line with recent findings on partisan differences in Covid-19 health behaviors (Allcott et al. 2020; Gadarian, Goodman and Pepinsky 2020), Democratic respondents expressed greater willingness to engage in all three measures than Republicans. Except for face-covering, the partisan differences in compliance willingness were sizeable and statistically significant. However, our analysis shows that both Democrats and Republicans are responsive to strict policy recommendations made by a either female or male governor.

Table A4: Treatment Assignment by In/ Out Partisan Groups

	No Strict	Strict Recommendations	Total
In-Partisan	193	184	377
Out-Partisan	159	161	320
Total	352	345	697

	Male Leader Treatment	Female Leader Treatment	Total
In-Partisan	194	183	377
Out-Partisan	166	154	320
Total	360	337	697

D. Balance Test

Table A5: Balance Tests for Treatment Conditions

	Dependent variable:			
	Female/Strict	Female/No strict	Male/Strict	Male/No strict
	(1)	(2)	(3)	(4)
Age	−0.008	−0.001	0.002	0.007
	(0.006)	(0.005)	(0.005)	(0.005)
Female	0.140	0.251	−0.066	-0.305^{*}
	(0.187)	(0.184)	(0.180)	(0.182)
Education	−0.073	−0.023	0.053	0.036
	(0.074)	(0.072)	(0.071)	(0.072)
Income	−0.008	−0.006	−0.0002	0.013
	(0.044)	(0.044)	(0.043)	(0.044)
White	−0.212	0.014	0.014	0.193
	(0.208)	(0.210)	(0.209)	(0.217)
Married	−0.146	0.026	0.120	−0.012
	(0.208)	(0.203)	(0.201)	(0.204)
Intercept	−0.362	-1.103^{***}	-1.376^{***}	-1.575^{***}
	(0.395)	(0.398)	(0.396)	(0.405)
Observations	688	688	688	688
Log Likelihood	-370.925	-384.600	-394.321	-386.394
Akaike Inf. Crit.	755.849	783.200	802.642	786.788

Note: *p<0.1; **p<0.05; ***p<0.01

References

Allcott, Hunt, Levi Boxell, Jacob Conway, Matthew Gentzkow, Michael Thaler and David Y Yang. 2020. "Polarization and public health: Partisan differences in social distancing during the Coronavirus pandemic." *NBER Working Paper* (w26946).

Gadarian, Shana Kushner, Sara Wallace Goodman and Thomas B Pepinsky. 2020. "Partisanship, health behavior, and policy attitudes in the early stages of the COVID-19 pandemic." *SSRN (preprint), Posted March* 27.

Krupnikov, Yanna and Nichole M. Bauer. 2014. "The Relationship Between Campaign Negativity, Gender, and Campaign Context." *Political Behavior* 36(1):167–188.
